# *In Vitro* Evaluation of ESE-15-ol, an Estradiol Analogue with Nanomolar Antimitotic and Carbonic Anhydrase Inhibitory Activity

**DOI:** 10.1371/journal.pone.0052205

**Published:** 2012-12-27

**Authors:** Barend Andre Stander, Fourie Joubert, Chingkuang Tu, Katherine H. Sippel, Robert McKenna, Annie Margaretha Joubert

**Affiliations:** 1 Department of Physiology, University of Pretoria, Pretoria, Gauteng, South Africa; 2 Department of Biochemistry, Bioinformatics and Computational Biology Unit, University of Pretoria, Pretoria, Gauteng, South Africa; 3 Department of Biochemistry and Molecular Biology, College of Medicine, University of Florida, Gainesville, Florida, United States of America; 4 Baylor College of Medicine, Houston, Texas, United States of America; 5 Department of Pharmacology and Therapeutics, University of Florida, Gainesville, Florida, United States of America; Wayne State University School of Medicine, United States of America

## Abstract

Antimitotic compounds are still one of the most widely used chemotherapeutic anticancer drugs in the clinic today. Given their effectiveness against cancer it is beneficial to continue enhancing these drugs. One way is to improve the bioavailability and efficacy by synthesizing derivatives that reversibly bind to carbonic anhydrase II (CAII) in red blood cells followed by a slow release into the blood circulation system. In the present study we describe the *in vitro* biological activity of a reduced derivative of 2-ethyl-3-O-sulphamoyl-estradiol (2EE), 2-ethyl-3-O-sulphamoyl-estra-1,3,5(10),15-tetraen-17-ol (ESE-15-ol). ESE-15-ol is capable of inhibiting carbonic anhydrase activity in the nanomolar range and is selective towards a mimic of carbonic anhydrase IX when compared to the CAII isoform. Docking studies using Autodock Vina suggest that the dehydration of the D-ring plays a role towards the selectivity of ESE-15-ol to CAIX and that the binding mode of ESE-15-ol is substantially different when compared to 2EE. ESE-15-ol is able to reduce cell growth to 50% after 48 h at 50–75 nM in MCF-7, MDA-MB-231, and MCF-12A cells. The compound is the least potent against the non-tumorigenic MCF-12A cells. *In vitro* mechanistic studies demonstrate that the newly synthesized compound induces mitochondrial membrane depolarization, abrogates the phosphorylation status of Bcl-2 and affects gene expression of genes associated with cell death and mitosis.

## Introduction

The clinical usefulness of antimitotic compounds that interfere with microtubule dynamics via the colchicine binding site, including 2-methoxyestradiol (2ME), chalcones and combretastatins, are currently under investigation [1]. Mediocre biopharmaceutical properties such as short half-life and low bioavailability of 2ME have prompted the research and development of estradiol analogues with improved *in vivo* efficacy [2], [3], [4], [5], [6]. The addition of a sulfamate group at position 3 of the estradiol backbone is known to improve the bioavailability of estradiol analogues [7], [8], [9]. The sulfamate group allows the compounds to bind the carbonic anhydrase II (CAII) of red blood cells in a reversible manner [10]. This allows the bypass of first pass metabolism due to the slow release into the blood stream from CAII, resulting in increased bioavaliability [7], [9].

An increased acidotic environment surrounding tumours are described to be the result of several metabolic alterations including increased glycolysis and lactate formation, and up regulation of extracellular carbonic anhydrase IX (CAIX) and carbonic anhydrase XII (CAXII) protein expression [11]. The conversion of carbon dioxide and water to carbonic acid by CAIX contributes to the acidification of the extracellular microenvironment [10], [12]. The acidic extracellular environment associated with tumors in turn promotes the expression of proteinases that contribute to invasion and metastasis [13], [14]. Compounds capable of selectivity inhibiting CAIX and therefore curtailing carbonic acid formation via CAIX over expression associated with many cancers is a strategy that can be employed to repress invasion and metastasis. A previous study identified 2-ethyl-3-O-sulphamoyl-estra-1,3,5(10),15-tetraen-17-one (ESE-15-one) as an antimitotic compound [2]. The present study describes the *in vitro* biological activity of a more potent reduced derivative of ESE-15-one, 2-ethyl-3-O-sulphamoyl-estra-1,3,5(10),15-tetraen-17-ol (ESE-15-ol). The carbonic anhydrase kinetics of ESE-15-ol on wild-type CAII and a CAIX mimic (CAII A65S N67Q) as well as docking poses of ESE-15-ol compared to its saturated D-ring derivative, 2-ethyl-3-O-sulphamoyl-estradiol are described (2EE) [15]. The *in vitro* effects of ESE-15-ol on cell growth, morphology, cell cycling and mitochondrial membrane potential in three different breast cell lines, including the metastatic MDA-MB-231, tumorigenic MCF-7 (estrogen receptor positive) and non-tumorigenic MCF-12A are described. ESE-15-ol was most potent against the MDA-MB-231 cells, therefore the effects of ESE-15-ol on Bcl-2 phosphorylation and global gene expression were analyzed in the MDA-MB-231 cell line.

The data in this paper suggest that ESE-15-ol is an antimitotic compound with potential anti-carbonic anhydrase IX activity that is more selective towards inhibiting growth in tumorigenic and metastatic MCF-7 and MDA-MB-231 cells when compared to non-tumorigenic MCF-12A cells. Furthermore, the data also suggests that ESE-15-ol is able to induce apoptosis by disrupting Bcl-2 dynamics in MDA-MB-231 cells.

## Materials and Methods

### Materials

Heat-inactivated fetal calf serum (FCS), sterile cell culture flasks, and plates were obtained through Sterilab Services (Kempton Park, Johannesburg, South Africa). Dulbecco’s minimum essential medium Eagle (D-MEM), penicillin, streptomycin, and fungizone were purchased from Highveld Biological (Pty) Ltd. (Sandringham, South Africa). A primary anti-tubulin alpha antibody from IMGENE (Alexandria, VA, USA) (cat no. IMG-80196) was purchased from BIOCOM biotech (Pty) Ltd. (Clubview, South Africa). The Alexa Fluor_ 488, anti-mouse IgG H+L secondary antibody from Invitrogen (Carlsbad, CA, USA) (cat no. A21202) was purchased from The Scientific Group (Johannesburg, South Africa). The Mitocapture™ apoptosis detection kit from BioVision Inc. (Mountain View, California, USA) was purchased from BIOCOM biotech (Pty) Ltd. (Pretoria, Gauteng, South Africa). The FlowCellect Bcl-2 Activation Dual Detection Kit was purchased from Millipore Corporation (Billerica, Massachusetts, USA). Qiagen’s RNeasy kit and RNase-free DNase were purchased from Southern Cross Biotechnology (Pty) Ltd. (Cape Town, South Africa). The Nanodrop, an Axon Genepix 4000B Scanner and Agilent’s Sure- Hyb chambers at the African Centre of Gene Technology (ACGT) Microarray Facility (University of Pretoria, Pretoria, South Africa) were purchased from Inqaba Biotechnical Industries (Pty) Ltd. (Pretoria, SA), Molecular Devices Corporation, (Sunnyvale, CA, USA) and Agilent Technologies (Pty) Ltd. (Palo Alto, CA, USA), respectively. The fluorescence activated cell sorting (FACS) FC500 System flow cytometer equipped with an air-cooled argon laser excited at 488nm was purchased from Beckman Coulter South Africa (Pty) Ltd. (Pretoria, South Africa). Agilent’s 44k 60-mer human oligo slides, Low RNA Input Fluorescent Linear Amplification Kit, 2× GEx Hybridization Buffer HI-RPM, Gene Expression (GE) Wash Buffer 1 and 2 and the Stabilization and Drying Solution were purchased from Agilent Technologies (Pty) Ltd. (Palo Alto, CA, USA). All other chemicals were of analytical grade and were purchased from Sigma Chemical Co. (St. Louis, MO, USA).

### Carbonic Anhydrase IX and II Kinetics

The inhibition of the catalyzed exchange of ^18^O between CO_2_ and water as measured by membrane-inlet mass spectrometry was used to determine the inhibition constants (K_i_) of ESE-15-ol on CAII and a CAIX mimic [16]. Membrane inlet mass spectrometry measures the rate of depletion of ^18^O from species of CO_2_. For carbonic anhydrase, the reaction velocity (R_1_/[E]) is for the rate of exchange between ^18^O-labeled carbonate to carbon dioxide and zinc-bound ^18^O-labeled hydroxide. The weighted average of the tight-binding inhibition constant K provides the K_i_ and is calculated using the Henderson method for tight-binding inhibitors [17].
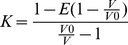

*V*_0_ is the reaction velocity (R1/[E]) observed in the absence of an inhibitor.

*V* is the reaction velocity (R_1_/[E]) observed at the concentration of an inhibitor.

*E* is the active enzyme concentration (7.3 nM).

Experiments were performed at 25°C in 0.1 M HEPES, pH7.4, 10 mM total carbonate concentration and inhibitor concentrations ranged up to 8 µM.

The pH of the extracellular growth medium of confluent MDA-MB-231 cells exposed to Desferoxamine (DFO), ESE-15-ol and a combination of DFO and ESE-16 for 24 h was measured in order to evaluate the ability of ESE-15-ol to prevent extracellular acidification by inhibiting CAIX activity [18]. An Orion 3 Star Benchtop pH meter from Thermo Fisher Scientific (Waltham, Massachusetts, USA) was used for the pH measurement of extracellular growth medium.

### Molecular Modeling

All molecular modeling studies were performed on an Intel I7 920 running Ubuntu 9. Receptor and ligand preparation was carried out by utilizing ACD/ChemSketch, Chimera, Reduce, VEGA 2.2.0 and AutoDockTools4 1.5.4 as described by Stander *et al.* (2011) [2], [19], [20], [21], [22]. Docking studies were carried out by Autodock Vina with a bounding box that encompassed the entire protein. Exhaustiveness was set to 35 with the rest of the parameters set on default [23]. An ensemble docking study was performed by docking ESE-15-ol and 2EE into the CAII (2gd8, 3bet, 3d8w, 3d9z, 3daz, 3dd0, 3oim, 3oku, 3x7t), wild-type CAIX (3iai, chains A-D) and the CAIX mimic (3dbu, 3dc3, 3dcc, 3dcs, 3dcw, 3oik, 3oil, 3okv) receptors. The lowest energy conformation was selected and the RMSD for 2EE was calculated with the crystal poses of 3EE bound to CAII (3oim) and CAIX mimic (3oil).

### Biology, Materials and Methods, Cell Culture and Maintenance

MDA-MB-231 (estrogen receptor negative) tumorigenic and metastatic breast cancer cells and MCF-7 (estrogen receptor positive) breast cancer cells were cultured in DMEM and supplemented with 10% heat-inactivated FCS (56°C, 30 min), 100 U/ml penicillin G, 100 µg/ml streptomycin and fungizone (250 µg/l). MCF-12A maintenance medium consisted of a 1∶1 mixture of DMEM and Ham’s-F12 medium, 20 ng/ml epidermal growth factor, 100 ng/ml cholera toxin, 10 µg/ml insulin and 500 ng/ml hydrocortisone, supplemented with 10% heat-inactivated FCS (56°C, 30 min), 100 U/ml penicillin G, 100 µg/ml streptomycin and fungizone (250 µg/l). ESE-15-ol was dissolved in dimethyl sulphoxide (DMSO). The final concentration of DMSO did not exceed 0.05% in cell culture. Experiments were conducted in 96-well, 6 well plates or 25 cm^2^ cell culture flasks. Exponentially growing cells were seeded at 5000 and 250 000 cells per well for 96-well, 6 well plates respectively. Cells were seeded at 750 000 cells per 25 cm^2^ cell culture flask. After a 24 h incubation period at 37°C to allow for cell adherence, cells were exposed to the compounds including the vehicle- control and incubated for 12 h, 24 h or 48 h at 37°C.

### Antiproliferative Assays–crystal Violet

Quantification of fixated monolayer cells was spectrophotometrically determined employing crystal violet as a DNA stain. Staining cell nuclei of fixed cells with crystal violet allows for rapid, accurate and reproducible quantification of cell number in cultures grown in 96-well plates [24], [25]. Dose-dependent studies were carried out in order to determine the growth inhibitory effect on the various cell lines of the newly synthesized compounds. The growth inhibitory effect was calculated as described by the National Cancer Institute in order to compare the growth inhibition induced by the compounds on the various cell lines [26]. The vehicle-treated control for each cell line was normalized to 100%.

### Immunohistochemistry of Tubulin and Nucleus

Confocal microscopy was employed to observe the effects of the new compounds on the cytoskeletal microtubule architecture of control and treated MDA-MB-231 cells. Cells were fixated with glutaraldehyde and alpha-tubulin was immunostained with anti-alpha tubulin antibodies. Anti-alpha tubulin antibodies were counter-stained with an Alexa-488 fluorescent probe and the nucleus was counter-stained with 4′,6-diamidino-2-phenylindole (DAPI). Stained cells were viewed with a Zeiss 510 META confocal laser microscope and figures were generated with Zeiss’ ZEN 2009 software.

### Analysis of Cell Cycle

Flow cytometry was employed to measure the DNA content of exposed and control cells in order to monitor the effect on cell cycle progression of MDA-MB-231 cells. Analysis was conducted by ethanol fixation and propidium iodide staining of cells. Propidium iodide was used to stain the nucleus in order to determine the amount of DNA present. Data from at least 10 000 cells was captured with CXP software (Beckman Coulter South Africa (Pty) Ltd). Cell cycle distributions were calculated with WEASEL version 3.0 software (F. Battye, Walter and Eliza Hall Institute (WEHI), Melbourne, Australia) by assigning relative DNA content per cell to sub-G_1_, G_1_, S and G_2_/M fractions. Time-dependent studies were carried out at intervals of 12 h, 24 h and 48 h.

### Mitochondrial Membrane Potential Detection

Mitochondrial membrane potential was monitored using Mitocapture™ by means of flow cytometry. Mitocapture™ is a cationic dye that accumulates and aggregates in the mitochondria of healthy cells, providing a bright red fluorescence [27]. In apoptotic cells, MitoCapture™ cannot aggregate in the mitochondria due to the altered mitochondrial membrane potential and thus remains in the cytoplasm in its monomer form, generating a green fluorescence [27]. After the 24 h exposure to ESE-15-ol and the vehicle (DMSO 0.05%), cells were trypsinized and 500 000 cells were incubated with 1 ml of the MitoCapture™ reagent for 15 min. The green fluorescence of the dye was measured with a fluorescence activated cell sorting (FACS) FC500 System flow cytometer (Beckman Coulter SA (Pty) Ltd.) in the FL1 channel. Data from at least 10 000 cells were analyzed with WEASEL version 3.0 software (F. Battye, Walter and Eliza Hall Institute (WEHI), Melbourne, Australia).

### Phosphorylation of Bcl-2 at Serine 70

Flow cytometry was employed to study the phosphorylation status of Bcl-2 at Ser 70, as well as the overall Bcl-2 protein expression in the MDA-MB-231 cell lines after 24 h exposure to ESE-15-ol compared to the vehicle-treated control. At 24 hours cells were trypsinized and 500 000 cells were centrifuged to discard the media. The cells were prepared as per the manufacturer’s instructions. Fluorescence of the FL1 (for measuring the Bcl-2 antibody) and FL3 (for measuring the pBcl-2, Ser 70) channel were measured with a FACS FC500 System flow cytometer. Data from at least 10 000 cells were analyzed with WEASEL version 3.0 software.

### Gene Expression

Agilent’s Human 1A Oligonucleotide Microarray 4x44k slides with more than 41,000 60-mer oligonucleotide human genes and transcripts were employed to collect genomic information on the ESE-15-ol mechanism of action in MDA-MB-231 cells. The detailed protocol for the human DNA microarray analysis has been reported in a previous study [28]. Briefly, total RNA was extracted from the MDA-MB-231 cells incubated with or without ESE-15-ol according to Qiagen’s RNeasy kit protocol. Five micrograms of mRNA from each biological replicate was used per array and all tests were performed in triplicate. Slides were scanned with the Axon Genepix 4000B Scanner. Spotfinding was performed using Genepix Pro 6.1 (Molecular Devices Corporation, Sunnyvale, CA, USA). Statistical analysis after spotfinding was conducted using Limma with the LimmaGUI interface [29], [30]. Background correction was done with the normal+exponential (Normexp) convolution model with an offset value was set to 25 [30], [31]. The Least squares linear model fit method was employed and the *P-*values were adjusted for multiple testing utilizing the Benjamini and Hochberg’s step-up method for controlling the false discovery rate [32]. Genes differentially expressed with a *P-*value of less than 0.05 were considered statistically significant. Biological interpretation and functional analysis of gene lists were performed by mapping differentially expressed genes to biochemical pathways and Gene Ontology (GO) categories using Gene Annotation Co-occurrence Discovery (GENECODIS) [33] GENECODIS is a web-based tool for finding sets of biological annotations that frequently appear together and are significant in a set of genes [33]. In order to determine common genes that were affected by the ESE-15-ol in MDA-MB-231 cells and 2ME in MCF-7 cells, differentially expressed gene lists were compared utilizing GeneVenn [34].

### Statistics

Data was obtained from 3 independent experiments. For crystal violet studies an *n*-value of 6 was obtained for each repeat. Obtained data was statistically analyzed for significance using a two-tailed Student’s *t*-test. Means are presented in bar charts, with T-bars referring to standard deviations. Measurement of Mitocapture-derived fluorescence was expressed as a ratio of the value measured for vehicle-treated exposed cells (relative fluorescence).

## Results and Discussion

### *In vitro* Carbonic Anhydrase Inhibition and Docking Analysis

**Figure 1:**
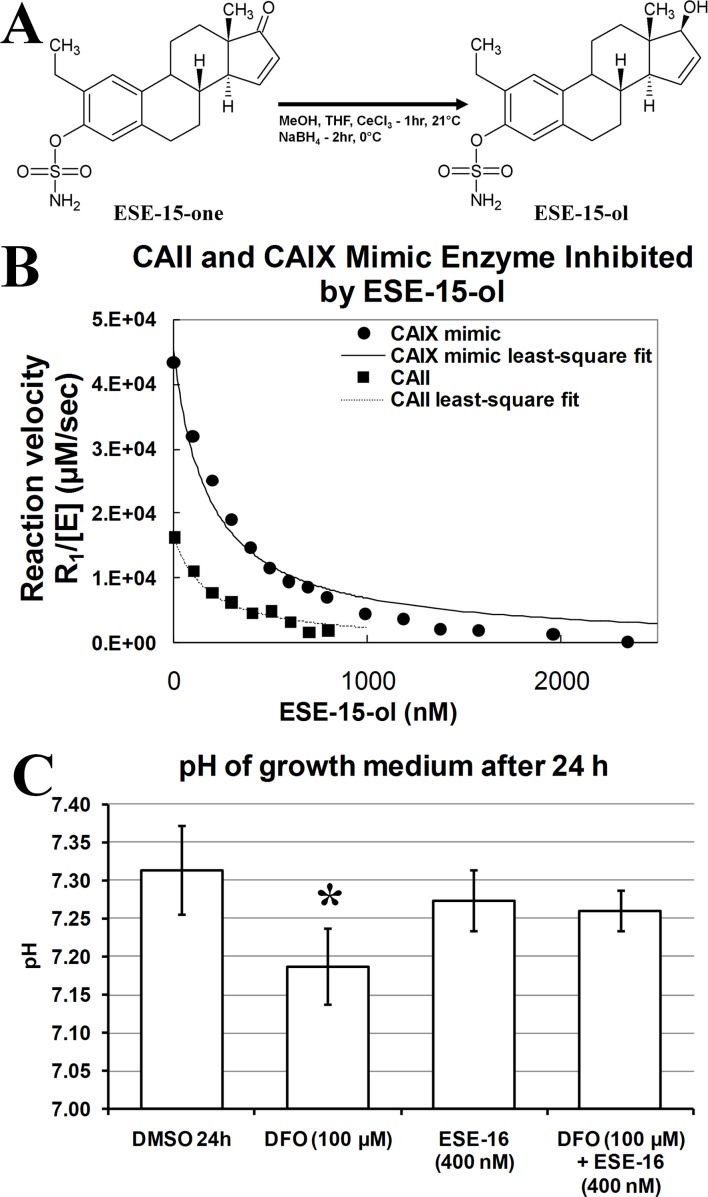
Synthesis of ESE-15-ol, inhibition of carbonic anhydrase activity by ESE-15-ol and the effects of ESE-15-ol on extracellular acidity. A) Synthesis of 2-ethyl-3-O-sulphamoyl-estra-1,3,5(10),15-tetraen-17-ol from 2-ethyl-3-O-sulphamoyl-estra-1,3,5(10),15-tetraen- 17-one. Reagents and conditions: (a) 2-ethyl-3-O-sulphamoyl-estra-1,3,5(10),15-tetraen-3-ol-17-one (0.11 mmol) and CeCl_3_ (0.12 mmol) in MeOH :THF (9∶2 *v*/*v*) at ambient temperature for 1hr. (b) After 1 hr, reaction was cooled to 0°C and NaBH_4_ (0.21 mmol) was added and stirred for 2 hr. B) Reaction velocity (R_1_/[E]) of wild-type CAII and a mimic of CAIX as determined by the catalysis of ^18^O exchange. Wild-type CAII K_i_ = 167±19 nM and CAIX mimic K_i_ = 89±23 nM, calculated using the Henderson method for tight-binding inhibitors. C) Changes in extracellular pH of confluent MDA-MB-231 cells treated with the CAIX inducer, DFO, and ESE-15-ol and a combination of DFO and ESE-15-ol. ESE-15-ol inhibited DFO-induced reduction in extracellular pH. * indicates a *t*-test *P-*value <0.05 for difference between vehicle-treated control and the DFO-treated samples.

**Figure 2:**
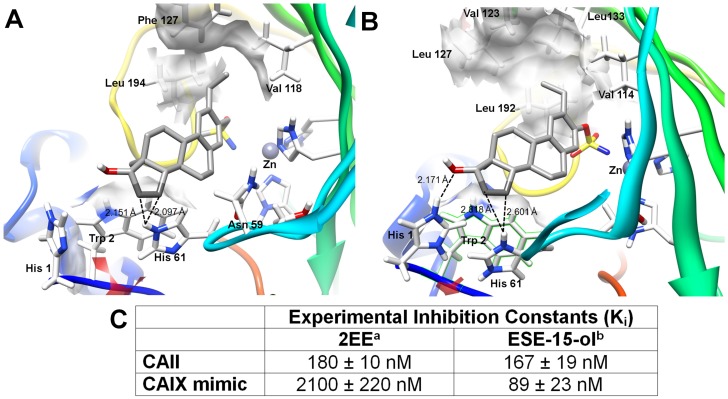
Docking and kinetics data of ESE-15-ol. Docking of compound 10 into a mimic of CAIX (A) and the wild-type isoform of CAIX (B) revealed that the double bond of ESE-15-ol at C15 and C16 may act as a nucleophile and interact with the electrophilic hydrogen of His 61. This interaction is posited to be specific of ESE-15-ol over 2EE and explain the isoform specificity of ESE-15-ol towards the CAIX mimic over CAII. Kinetics data (C) demonstrates that ESE-15-ol has an almost 2-fold higher affinity for the CAIX mimic when compared to the wild-type CAII. *^a^* Experimental inhibition constants were determined by Sippel *et al.* (2011) [35]. *^b^* The inhibition of the catalyzed exchange of ^18^O between CO_2_ and water as measured by membrane-inlet mass spectrometry was used to determine the experimental inhibition constants (K_i_) of ESE-15-ol [16]. A CA IX mimic was used as characterized previously by Genis *et al*. 2009 [15].

ESE-15-ol was synthesized (Figure 1A, Supporting Information S1 and S2) and assessed for carbonic anhydrase inhibition of the physiologically dominant CAII and a mimic of the tumor-associated CAIX using gas inlet mass spectroscopy. The accuracy of CAIX mimic as a model of wild-type CA provides a useful tool to test isozyme-specific CA IX inhibitors [15], [35]. Kinetics data indicated that ESE-15-ol has an almost 2-fold higher affinity for the CAIX mimic when compared to the wild-type CAII (Figures 1B and 2). This was surprising as the saturated D-ring derivative of ESE-15-ol, 2EE, is reported to have a 12-fold reduction in affinity for the CAIX mimic when compared to CAII [35]. 2EE was docked with Autodock Vina into various CAII receptors as well as receptors of the mimic of CAIX and the wild type CAIX. The lowest energy score for 2EE was obtained in 3oku (CAII, −9 kcal/mol) and 3dcs (CAIX mimic, −8.2 kcal/mol) with root means squared deviation (RMSD) values of 1.332 and 1.42 respectively compared to the crystal pose (Supporting Information S3). This indicated to us that the docking software was able to reproduce the crystal poses and relative binding energies reasonably well and would be useful in determining the possible binding mode and pose of ESE-15-ol into CAII, the wild-type CAIX and the CAIX mimic. Chain C of the wild-type CAIX (3iaia_C) yielded the lowest dock energy for 2EE (-9 kcal/mol). The 3oil crystal structure with 2EE bound to the CAIX mimic was superimposed onto 3iai_C (RMSD = 0.32) and the RMSD of 2EE docked into 3aia_C was 1.61 when compared to the crystal structure of 2EE in 3oil (Supporting Information S3). This indicates that the docking pose of 2EE into the wild-type CAIX is probably similar to that of 2EE into the CAIX mimic.

The lowest energy score for ESE-15-ol was when it was docked into 2oku (−9 kcal/mol), 3okv (−8.3 kcal/mol) and 3aia_C (−9.3 kcal/mol) for CAII, the CAIX mimic and the wild-type CAIX respectively. The docking pose of ESE-15-ol into CAII was similar to that of the docking pose of 2EE into CAII (Supporting Information S3). Sippel *et al.* (2011) reported that the inhibition constant (K_i_) for 2EE in CAII is 180±10 nM [35]. The present study reports that the K_i_ of ESE-15-ol in CAII is 167± nM (Figure 2), indicating that the binding modes of 2EE and ESE-15-ol into CAII are likely very similar. However, when we look at the binding pose of ESE-15-ol into the CAIX mimic and the wild-type CAIX we observe different interactions (Figure 2 and Supporting Information S3). Sippel *et al.* (2011) reported that 2EE has a hydrophobic interaction with Asn 62 at the D-ring and that the O17 hydroxyl form a weak hydrogen bond with a water that interacts with Gln 67 [35]. ESE-15-ol, however, appears to have very different interactions at the D-ring. In the CIAX mimic, the double bond of ESE-15-ol at C15 and C16 acts as a nucleophile and is able to interact with the electrophilic hydrogen of His 61 (Figure 2). This position also allows ESE-15-ol to have Van der Waals interactions with Trp 2 as well as Asn 59. ESE-15-ol also engages in hydrophobic Van der Waals contacts with Val 118, Leu 194 and Phe 127. In the wild-type CAIX, the His 1 residue is closer to the O17 hydroxyl group and can form a hydrogen bond while retaining the electrophilic, nucleophilic interaction between His 61 and the C15–C16 double bond of ESE-15-ol (Figure 2). Together these interactions may explain the isoform specificity of ESE-15-ol towards the CAIX mimic over CAII and also suggest that it may even be more specific towards the wild-type CAIX due to the potential His 1-O17 hydrogen bond interaction.

None of the cell lines in our study express CAIX under the conditions we tested the compound. However, CAIX protein expression can be induced in MDA-MB-231 cells by the iron chelator, DFO, in a manner that results in extracellular acidification due to CAIX over expression [18]. Confluent MDA-MB-231 cells were exposed to ESE-15-ol with and without DFO for 24 h. DFO treatment resulted in a statistically significant decrease in pH of the growth medium when compared to the DMSO control. Treatment of MDA-MB-231 cells in conjunction with ESE-15-ol (100 nM) prevented the acidification (Figure 1C), suggesting that ESE-15-ol does prevent extracellular acidification due to CAIX expression.

### Antiproliferative Activity of ESE-15-ol

**Figure 3:**
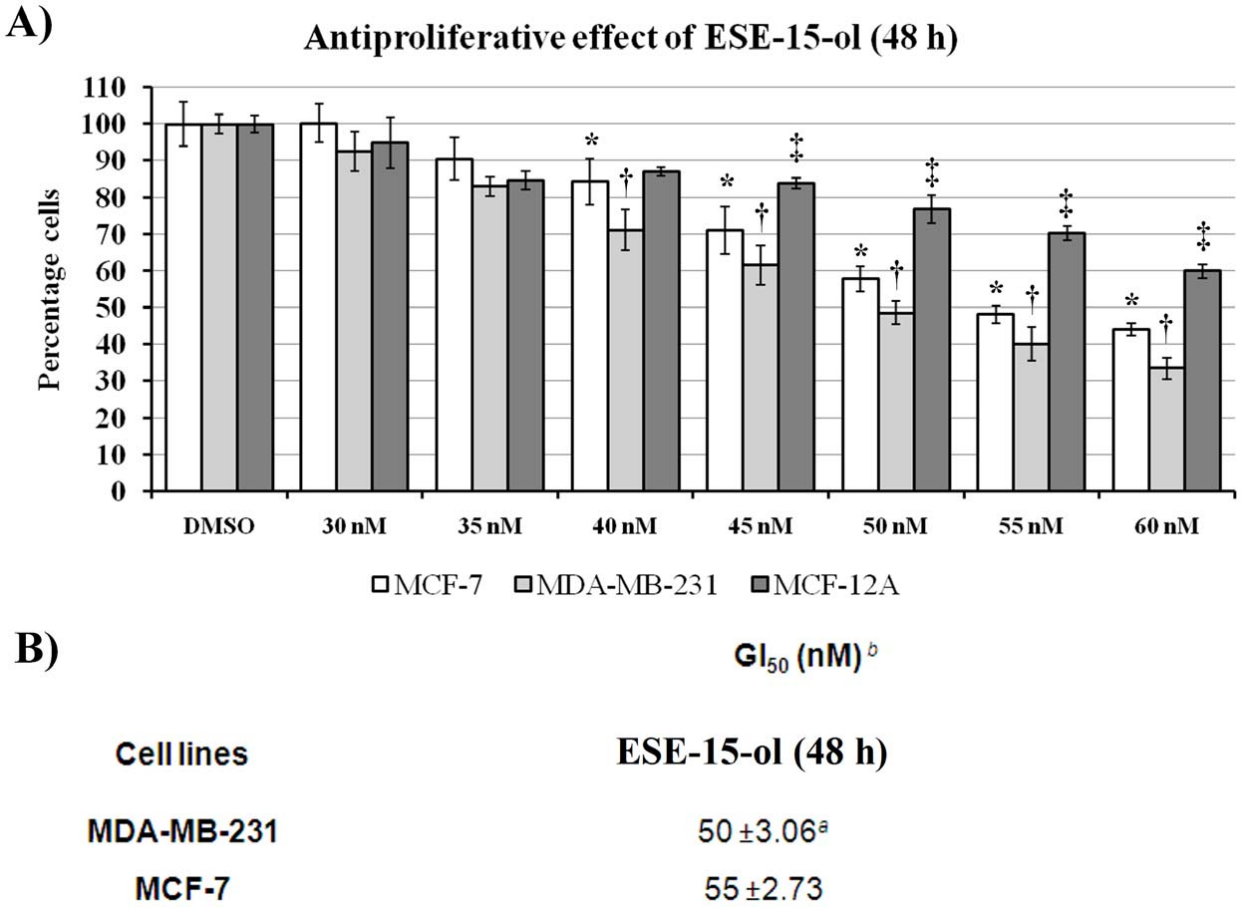
Antiproliferative effects of ESE-15-ol on MDA-MB-231, MCF-7 and MCF-12A cells after 48 h exposure A) Dose-dependent analysis of DNA content revealed a statistically significant 50% growth inhibitory effect (GI_50_) of malignant cell numbers after 48 h of exposure to be 55 nM of ESE-15-ol for in MCF-7 and 50 nM for the MDA-MB231. MCF-12A cells were the least affected at 50 nM of ESE-15-ol. *^a^* Values are presented as the average of three biological replicates (*N* = 6) ± SD. *^b^* The compound concentration required to inhibit cell proliferation by 50% was determined by exposing cells for 48 hours to test inhibitors. * indicates a *t*-test *P-*value <0.05 for growth inhibition between MCF-7 and MDA-MB-231 cells. † indicates a *t*-test *P-*value <0.05 for growth inhibition between MDA-MB-231 and MCF-12A cells. ‡ indicates a *t*-test *P-*value <0.05 for growth inhibition between MCF-12A and MCF-7 cells.

ESE-15-ol was screened for antiproliferative activity using crystal violet as a DNA stain as described by Berry *et al.* (1996) [36]. The assay was conducted on metastatic MDA-MB-231 breast cancer cells, tumorigenic MCF-7 breast cancer cells (estrogen receptor positive) and non-tumorigenic MCF-12A breast cells. ESE-15-ol reduced cell proliferation in a dose-dependent manner in all tested cell lines (Figure 3). The results indicated that ESE-15-ol has the lowest GI_50_ concentration for the metastatic MDA-MB-231 cells (50 nM). The data indicates that the MCF-12A cell line was the least affected of the three cell lines at 50 nM. It was therefore decided carry out all subsequent studies with the GI_50_ of MDA-MB-231 in order to determine the differential effects of this concentration across the different cell lines.

### Morphological Effects of ESE-15-ol on Tubulin Architecture

**Figure 4:**
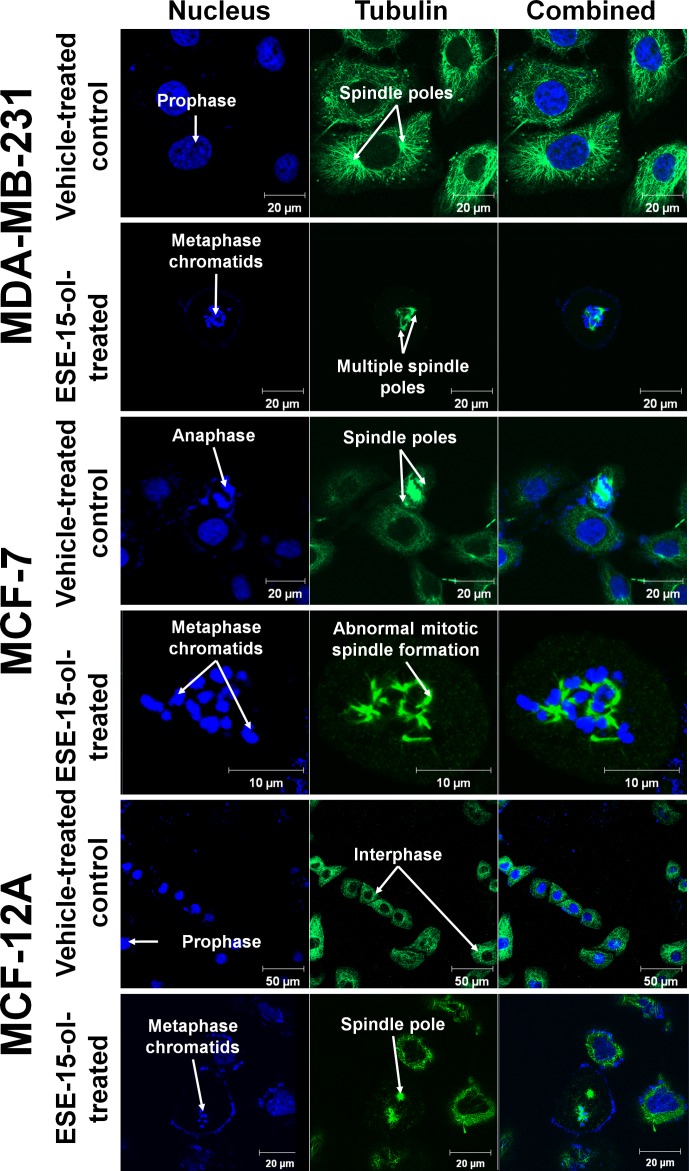
Vehicle-treated control and ESE-15-ol-treated cells stained with 4′,6-diamidino-2-phenylindole and Alexa-488 anti-tubulin after 24-h exposure. Vehicle-treated in MDA-MB-231, MCF-7 and MCF-12A cells show no signs of abnormal cell cycle completion formation. ESE-15-ol-treated cells show various degrees of abrogated cell cycling including abnormal mitotic spindle formation and multiple spindle pole formation during mitosis.

The cytoskeletal microtubule architecture of control and treated MDA-MB-231, MCF-7, and MCF-12A cells were monitored via confocal microscopy after 24 h exposure. The formation of multiple spindle poles, as well as abnormal mitotic spindle formation during mitosis was observed in ESE-15-ol-treated (50 nM) MDA-MB-231, MCF-7, and MCF-12A cells (Figure 4). Vehicle-treated cells were unaffected (Figure 4). Abrogation of spindle formation during mitosis is a property of antimitotic agents, confirming that ESE-15-ol is an antimitotic compound that interferes with the microtubule dynamics in actively dividing cells.

### Cell Cycle Analysis

**Figure 5:**
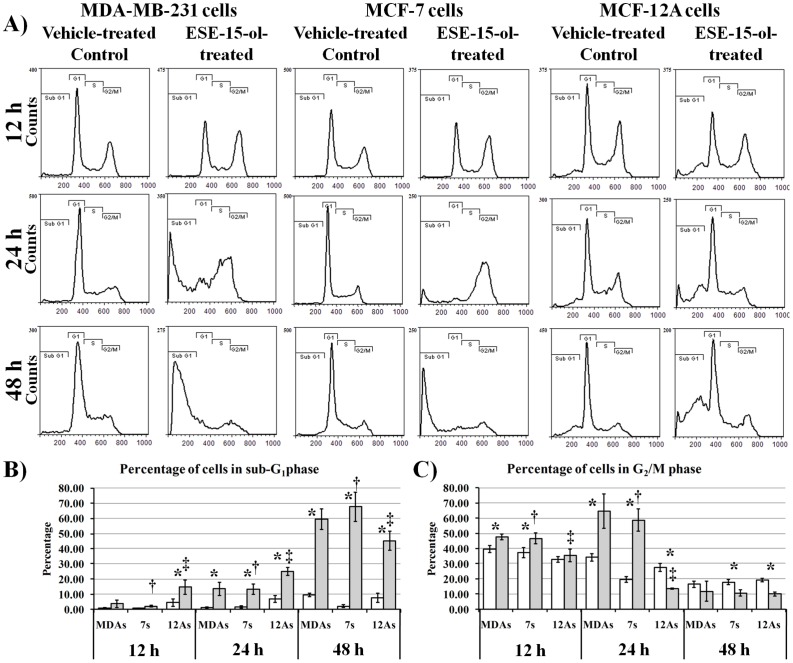
Effects of ESE-15-ol on cell division over time. A) Cell cycle histograms of vehicle-treated and ESE-15-ol-treated MDA-MB-231, MCF-7 and MCF-12A cells after 12 h, 24 h and 48 h exposure. B) Time-dependent change of vehicle-treated and ESE-15-ol-treated MDA-MB-231, MCF-7 and MCF-12A cells in sub G_1_ B) Time-dependent change of vehicle-treated and ESE-15-ol-treated MDA-MB-231, MCF-7 and MCF-12A cells in mitosis (G_2_/M). A gradual increase in the sub-G_1_ fraction is observed over time in ESE-15-ol-treatment across all cell lines, indicating increased cell death. After 48 h, the MCF-12A cells were less affected when compared to the MDA-MB-231 and MCF-7 cells. * indicates a *t*-test *P-*value <0.05 for difference between vehicle-treated control and ESE-15-ol-treated samples. † indicates a *t*-test *P-*value <0.05 for difference between MCF-7 and MCF-12A ESE-15-ol-treated samples. ‡ indicates a *t*-test *P-*value <0.05 for difference between MCF-12A and MDA-MB-231 ESE-15-ol-treated samples.

To determine the effect that ESE-15-ol has on cell cycle progression the DNA content of cells was measured after 12 h, 24 h and 48 h (Figure 5). The sub-G_1_ phase is indicative of cells undergoing cell death via DNA cleavage. A statistically significant time-dependent increase in the sub-G_1_ fraction of ESE-15-ol-treated cells was observed in all cell lines. However statistically the MCF-12A cell line was less affected by ESE-15-ol after 48 h (45.48%) when compared to MDA-MB-231 (58.41%) and MCF-7 (67.72%) (Figure 5B). In MDA-MB-231 and MCF-7 ESE-15-ol-treated cells there was an increase in the G_2_/M phase cells after 12 h and 24 h. After 48 h, the cells appeared to have exited the mitotic block and entered the sub-G_1_ phase. These results indicate that ESE-15-ol is able to induce a mitotic cell cycle arrest followed by apoptosis. It also indicates that the non-tumorigenic MCF-12A cells are less susceptible to apoptotic cell death after 48 h when compared to the tumorigenic MCF-7 and metastatic MDA-MB-231 cells.

### Mitochondrial Membrane Potential Analysis

**Figure 6:**
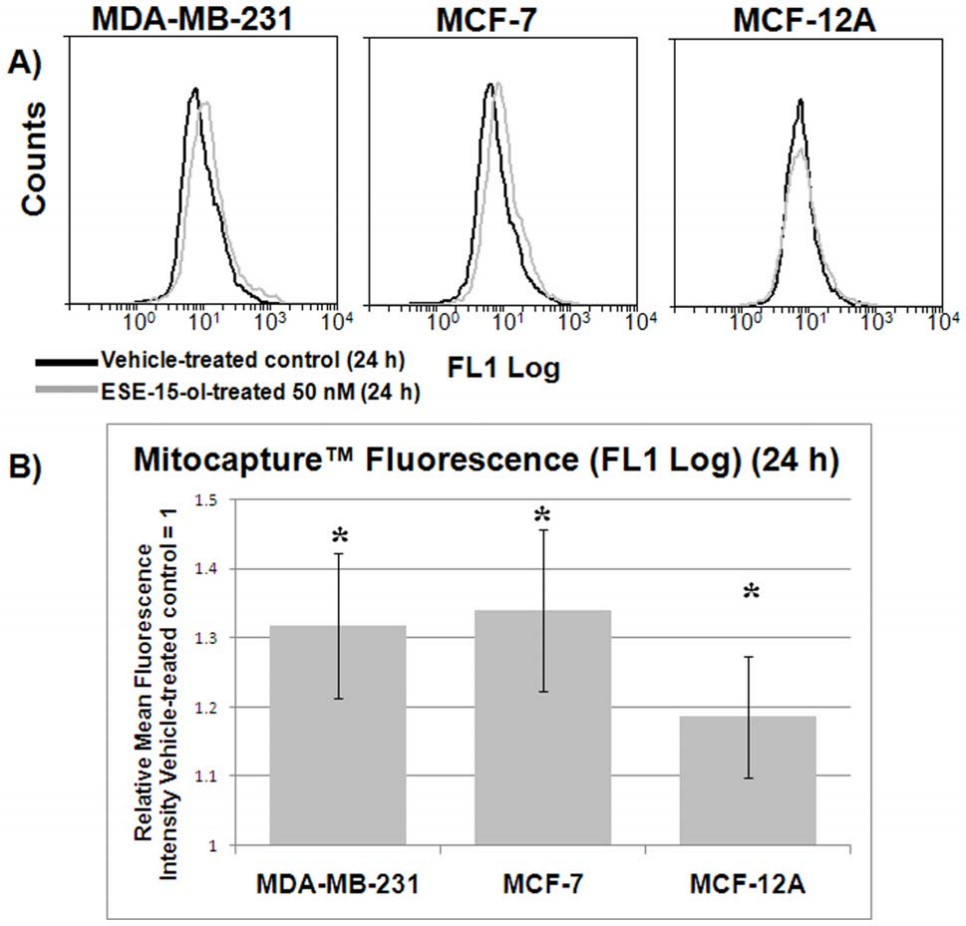
Effects of ESE-15-ol on mitochondrial membrane potential in MDA-MB-231, MCF-7 and MCF-12A cells after 24 h exposure. A) Flow cytometry histograms indicating relative fluorescence intensity for Mitocapture™ in the red channel (FL3 log). An increase in FL3 log indicates an increase in mitochondrial membrane depolarization and potential apoptosis. B) A statistically significant increase in mitochondrial membrane depolarization in the ESE-15-ol-treated cells compared to the vehicle-treated cells was observed across all three cell lines. * indicates a *P-*value <0.05 between vehicle-treated cells and ESE-16-treated cells.

Mitochondrial membrane potential was analyzed using MitoCapture via flow cytometry. In apoptotic cells the reagent cannot aggregate in the mitochondria due to the altered membrane potential, and remains monomeric and in the cytoplasm generating a green fluorescence [27]. There was a statistically significant increase of green fluorescence in the ESE-15-ol-treated cells across all the cell lines after 24 h exposure (Figure 6). These data suggests that apoptosis is one possible form of cell death being induced by ESE-15-ol.

### Phosphorylation of Bcl-2 at Serine 70

**Figure 7:**
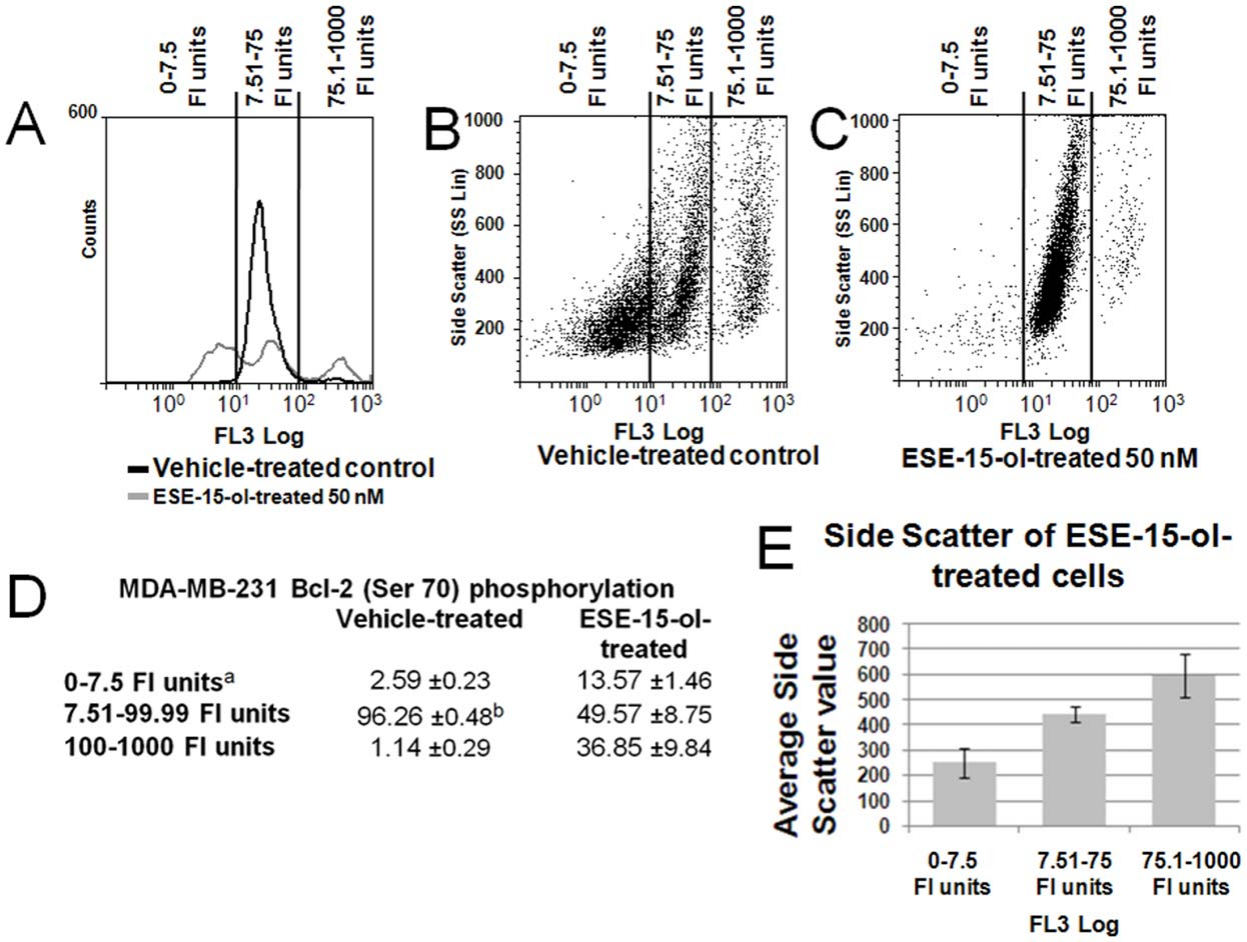
Effects of ESE-15-ol on Bcl-2 phosphorylation in MDA-MB-231 cells after 24 h exposure. A) Flow cytometry histogram of Bcl-2 (Ser 70) relative quantity (FL3-log) in vehicle-treated and ESE-15-ol-treated (50 nM) MDA-MB-231 cells after 24 h exposure. Flow cytometry dot-plot of Bcl-2 (Ser 70) (FL3-log) relative quantity vs relative cellular complexity (SS lin) in B) vehicle-treated or C) ESE-15-ol-treated MDA-MB-231 cells. D) Percentage of cells in the FI unit ranges of 0–7.5, 7.51–99.99 and 100–1000 as an indication of the quantity of Bcl-2 (Ser 70) phosphorylation per cell. E) Bar-chart demonstrating the differences in cell size with different amounts of Bcl-2 (Ser 70) per cell. Means of three biological replicates are presented in bar charts. No differences in the overall Bcl-2 expression was observed (data not shown). ^a^ The fluorescence intensity of the FL3 filter is indicated by “FI units”. ^b^ Values are presented as the average ± SD.

The FlowCellect Bcl-2 Activation Dual Detection Kit (Millipore) uses two antibodies to measure the abundance of Bcl-2 protein expression generally and the abundance of Bcl-2 phosphorylated at Ser 70 specifically. Our studies indicate that overall Bcl-2 expression is unchanged in ESE-15-ol-treated MDA-MB-231 cells compared to the vehicle control (data not shown). However, results indicate that the phosphorylation status of Bcl-2 in ESE-15-ol-treated MDA-MB-231 cells is very different. There were fewer cells in the fluorescence intensity (FI) unit range of 7.51–100 in ESE-15-ol-treated cells when compared to the vehicle-treated cells (Figures 7A and 7D). The FI unit range of 7.51–100 corresponds to phosphorylation status of Bcl-2 at Ser 70 for >90% of cells in the vehicle-treated sample while it was only ±50% for the vehicle-treated cells (Figures 7A and 7D). In ESE-15-ol-treated cells the population shifted towards either an increase or decrease in phosphorylation (Figures 7A and 7D). Further analyses of the dot-plot data indicated that ESE-15-ol-treated cells with increased Bcl-2 phosphorylation tended to have a higher side-scatter (SS Lin) signal (Figures 7B, 7C and 7E). Side scatter depends on the inner complexity of the particle. For example, an increase in the amount of DNA in a cell would correlate to an increased side-scatter signal. The data suggests that ESE-15-ol-treated cells with increased inner complexity also have an increase in Bcl-2 Ser 70 phosphorylation. Bcl-2 is a key regulator of mitochondrial membrane potential and mitochondrial mediated apoptosis induction. An increase in the phosphorylation of Bcl-2 on Ser 70 alone prevents apoptosis while multi-site phosphorylation at residues Ser 70, Trp 69 and Ser 87 is associated with a G_2_/M block in MCF-7 and MDA-MB-231 cells and leads to apoptotic induction [37]. Dephosphorylation at Ser 70 or an overall decrease in the protein expression of Bcl-2 is also associated with apoptosis [37]. These results suggest that ESE-15-ol is able to abrogate the balance of Bcl-2 phospohorylation in a manner that promotes apoptosis via intrinsic pathways.

### Gene Expression Analysis

Agilent’s Human 1A Oligonucleotide Microarray was employed to collect transcriptional information to determine ESE-15-ol’s mechanism in MDA-MB-231 cells. Genes that were considered statistically significantly differentially expressed (adjusted *P*-value <0.05) and up regulated or down regulated in ESE-15-ol-treated MDA-MB-231 cells are summarized in Supporting Information S4. The 399 genes that were differentially expressed in ESE-15-ol-treated MDA-MB-MB231 cells were compared to 775 genes differentially expressed in 2ME-treated MCF-7 cells [28], [34]. The differentially expressed genes were mapped to genes associated with apoptosis, autophagy, metastasis, cell cycle and stress response proteins., They include kinases, phosphatases, epigenetic and chromatin modifiers, structural proteins, transcription factors and nuclear proteins, RAS and RAB related proteins, and proteosomal components (Supporting Information S4 and S5). A total of 113 genes were found to be differentially expressed in both ESE-15-ol-treated MDA-MB-231 cells and 2ME-treated MCF-7 cells. These results indicate that there is likely a common mechanism of action between the two compounds.

Of particular interest are those genes responsive to oxidative stress including heme oxygenase (decycling) 1, spermine oxidase, and the Bcl-2 binding 3 component (BBC3/PUMA) protein and stress-related proteins. Heme oxygenase mRNA expression is increased by the generation of reactive oxygen species such as hydrogen peroxide [38]. Spermine oxidase upregulation is known to result in antiproliferative effects produced from the oxidative stress of spermine to spermidine conversion [39], [40]. An altered redox status also leads to the expression of the pro-apoptotic BBC3/PUMA protein [41]. These results suggest that the ESE-15-ol cytotoxic effect may be driven by mitochondrial mediated apoptotic induction linked to the redox status of the cell.

Additionally the transcripts differentially expressed in both 2ME- and ESE-15-ol-treated cells included cell cycle related genes and several histone cluster H3 proteins. Previous studies demonstrated that mitotic checkpoint proteins play an important role in regulating chromatin remodeling and vice versa [42], [43]. Histone cluster H3 proteins help regulate the mitotic checkpoint via the tension sensing mechanism of the spindle assembly checkpoint [42], [43]. Also, the “remodel the structure of chromatin” (RSC) chromatin-remodeling complex and a functioning cdc5 protein are needed for the timely exist from mitosis in actively dividing cells [44]. Cdc5 is the yeast homologue of polo-like kinase 1 (PLK1) and is interestingly down regulated in ESE-15-ol-treated cells (Supporting Information S5). This data suggests that ESE-15-ol interferes with the normal crosstalk between the chromatin remodeling and the spindle assembly checkpoint apparatus. Further studies are needed to determine the exact mechanism of action and the implications of this interference.

### Conclusions

In the present study, we performed the synthesis and *in vitro* biological evaluation of 2-ethyl-3-O-sulphamoyl-estra-1,3,5(10),15-tetraen–17-ol, a new antimitotic estradiol compound. Kinetics studies indicated that ESE-15-ol is more selectively towards inhibiting a CAIX mimic than wild-type CAII. Docking studies suggest that the selectivity is due to the double bond in the D-ring that is capable of interacting with His 61 of the cancer-associated CAIX. *In vitro* studies with a known inducer of CAIX expression in MDA-MB-231 cells suggest that ESE-15-ol can prevent extracellular acidification because of CAIX expression, suggesting that the compound has the potential to curtail metastatic processes associated with acidotic microenvironmental conditions in tumors. ESE-15-ol was able to reduce cell growth to 50% of the vehicle-treated control in the nanomolar range and was more potent against metastatic MDA-MB-231 cells and tumorigenic MCF-7 cells when compared to the non-tumorigenic MCF-12A cell line. Confocal microscopy demonstrated the antimitotic effects of ESE-15-ol on actively dividing cells. Cell cycle analyses further demonstrated that ESE-15-ol is able to block cells in the G_2_/M phase after 12 h exposure with cell death induced after 48 h.

Apoptotic cell death through mitochondrial membrane potential depolarization via the intrinsic pathway has been confirmed by flow cytometric analyses of Mitocapture fluorescence. Disruption of Bcl-2 phosphorylation as a result of ESE-15-ol exposure is likely to play a mechanistically relevant role in mitochondrial membrane potential depolarization. Also, the up regulation in the gene expression of the pro-apoptotic Bcl-2 binding protein (BBC3/PUMA) in both 2ME and ESE-15-ol-treated cells suggest a common mechanism of action between the two antimitotic compounds. Data from gene expression analyses suggest the involvement of ROS formation in inducing cell death as well as an interference in the crosstalk between the chromatin remodeling and mitotic spindle checkpoint apparatus in ESE-15-ol-treated cells. This study indicates that ESE-15-ol is a promising antimitotic anticancer drug that warrants further investigation.

## Supporting Information

Supporting Information S1 **Synthesis of ESE-15-ol.**
(DOCX)

Supporting Information S2 **Confirmation of purity and structure and via ^1^H NMR (400 MHz CDCl_3_** ***)*** **and mass spectrometry.**
(DOCX)

Supporting Information S3 **Crystal structures of 2EE (white) positioned in CAII (A and C) and the CAIX mimic (B).** The docking poses of 2EE into CAII (A, pink ligand) and into the CAIX mimic (B, yellow ligand) have an RMSD value of 1.332 and 1.42 respectively compared to the crystal pose. ESE-15-ol docked into CAII (C, pink ligand) show a close fit compared to the crystal pose of 2EE in CAII.
(DOCX)

Supporting Information S4 **Differentially expressed genes revealed by amplified cRNA microarray and bioinformatics analyses in MDA-MB-231 cells exposed to ESE-15-ol (24 h at 50 nM).**
(DOCX)

Supporting Information S5 **Common differentially expressed genes revealed by amplified cRNA microarray and bioinformatics analyses in MDA-MB-231 cells exposed 24 hours to 50 nM ESE-15-ol or MCF-7 cells exposed to 1 µM 2ME.**
(DOCX)
