# Supplementary material for: In Vitro Evaluation of ESE-15-ol, an Estradiol Analogue with Nanomolar Antimitotic and Carbonic Anhydrase Inhibitory Activity
Source: PLoS One. 2012 Dec 27;7(12):e52205. doi: 10.1371/journal.pone.0052205 (PMC3531393; doi:10.1371/journal.pone.0052205)
Supplement: Supporting Information S1 — Synthesis of ESE-15-ol. (DOCX) [file pone.0052205.s001.docx]

# Synthesis of 2-ethyl-3-O-sulphamoyl-estra-1,3,5(10),15-tetraen-3-ol-17-ol

## Materials and Methods

The synthesis of the estradiol analogue, C10, was outsourced to iThemba Pharmaceuticals (Pty) Ltd (Modderfontein, Gauteng, South Africa). All chemicals were purchased from Aldrich Chemical Co (St. Louis, MO, USA). Organic solvents of A.R. grade were used as supplied. Anhydrous *N,N*-dimethylformamide and *N,N*-dimethylacetamide were purchased from Aldrich and stored under a positive pressure of N_2_ after use. Tetrahydrofuran was distilled from sodium. Sulphamoyl chloride was prepared by an adaptation of the method of Appel and Berger and stored in a tightly sealed container in the fridge [[15](#_ENREF_15)]. Chromatography was performed on silica gel (70–230 mesh, Macherey Nagel). Thin layer chromatography was performed on Alugram® SIL G/UV_254_ aluminium backed plates (Macherey Nagel). Products were visualized with basic potassium permanganate solution. ^1^H NMR spectra were recorded in deuterated chloroform solution (unless otherwise indicated) with a Varian 400 NMR spectrometer at 400 MHz. Chemical shifts are reported in parts per million (ppm) relative to tetramethylsilane (TMS) as an internal standard. Compounds were synthesized according to scheme 1 in Figure 1. All the compounds were >90% pure and were analyzed by NMR.

## Synthesis

2-ethyl-3-O-sulphamoyl-estra-1,3,5(10),15-tetraen-3-ol-17-one (0.040 g, 0.11 mmol) was added to a solution of methanol (9.00 cm^3^) and tetrahydrofuran (2.00 cm^3^) at ambient temperature. Cerium (III) chloride heptahydrate (0.044 g, 0.12 mmol) was added and stirring was continued for 1 hour. The reaction mixture was cooled to 0 °C and sodium borohydride (0.008 g, 0.21 mmol) was added in several portions. After 3 hours, a saturated solution of ammonium chloride (5 cm^3^) was added and the aqueous reaction mixture was extracted with ethyl acetate (50 cm^3^). The organic phase was washed with water (10 cm^3^) and brine (10 cm^3^). The organic extract was dried over sodium sulfate, filtered and evaporated to give a yellow oil. Column chromatography (40 % ethyl acetate/hexane) afforded 2-ethyl-3-O-sulphamoyl-estra-1,3,5(10),15-tetraen-3-ol-17-ol (0.030g, 0.08 mmol, 75% yield) as a white foam. The compound is >95% pure with trace amounts of ethyl acetate, hexanes and acetone (Supplementary Information S2). The solid was stored in the freezer when not in use: *R_f_* 0.39 (3:2 hexane/ethyl acetate). ^1^H NMR (400 MHz, *CDCl_3_*) 7.20 (s, 1H), 7.09 (s, 1H), 5.94-5.85 (m, 1H), 5.79-5.70 (m, 1H), 4.93 (br s, 2H), 4.52 (br s, 1H), 2.95-2.78 (m, 2H), 2.69 (q, *J =* 7.6 Hz, 2H), 2.43 (s, 1H), 2.37-2.19 (m, 2H), 1.84-1.66 (m, 3H), 1.64-1.53 (m, 2H), 1.48-1.36 (m, 1H), 1.24 (s, 3H), 1.21 (t, *J =* 7.6 Hz, 3H).

**Purity**



**Supplementary Figure 1:** Confirmation of structure and purity via ^1^H NMR (400 MHz CDCl_3_)
