# Supplementary material for: In Vitro Evaluation of ESE-15-ol, an Estradiol Analogue with Nanomolar Antimitotic and Carbonic Anhydrase Inhibitory Activity
Source: PLoS One. 2012 Dec 27;7(12):e52205. doi: 10.1371/journal.pone.0052205 (PMC3531393; doi:10.1371/journal.pone.0052205)
Supplement: Supporting Information S2 — Confirmation of purity and structure and via 1H NMR (400 MHz CDCl3 ) and mass spectrometry. (DOCX) [file pone.0052205.s002.docx]

**Purity**



**Supplementary Figure 1:** Confirmation of structure and purity via ^1^H NMR (400 MHz CDCl_3_**)**

**Supplementary Figure 2: EIMS of ESE-15-ol obtained on a Thermo DFS with 70 eV**

**
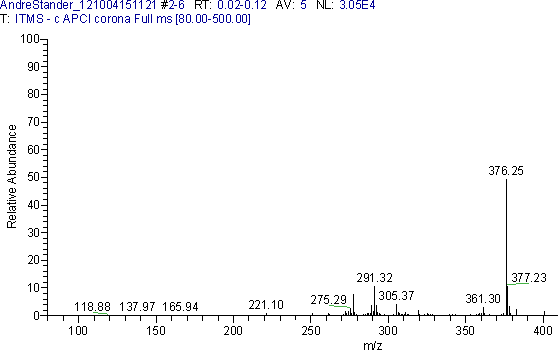
**

**Supplementary Figure 3: APCI-MS / MS^2^ (negative mode) of ESE-15-ol.**

| **Compound** | **Molecular**  **Formula** | **Molecular wt**  **(Theoretical)** | **Molecular wt (Mass Spectrometry)** |
| --- | --- | --- | --- |
| **ESE-115-ol** | **C_20_H_27_O_4_N_1_S_1_** | **377.49768** | **359.15527 (Supplementary Figure 2: [C_20_H_27_O_4_N_1_S_1_-H_2_O]^+^)**  **376.25 (Supplementary Figure 3: [C_20_H_27_O_4_N_1_S_1_-H]^-^)** |
