# Supplementary material for: In Vitro Evaluation of ESE-15-ol, an Estradiol Analogue with Nanomolar Antimitotic and Carbonic Anhydrase Inhibitory Activity
Source: PLoS One. 2012 Dec 27;7(12):e52205. doi: 10.1371/journal.pone.0052205 (PMC3531393; doi:10.1371/journal.pone.0052205)

# Supplementary information S2: Crystal structures of 2EE (white in A, C and D and cyan in B) positioned in CAII (A and C) and the CAIX mimic (B and D). The docking poses of 2EE into CAII (A, pink ligand) and into the CAIX mimic (B, yellow ligand) have an RMSD value of 1.332 and 1.42 respectively compared to the crystal pose. ESE-15-ol docked into CAII (C, pink ligand) shows a close fit compared to the crystal pose of 2EE in CAII. The docking pose of ESE-15-ol into the CAIX mimic diverges from 2EE, allowing an interaction between the nucleophilic double bond at C15 and C16 and the electrophylic hydrogen of His 61(D).

#
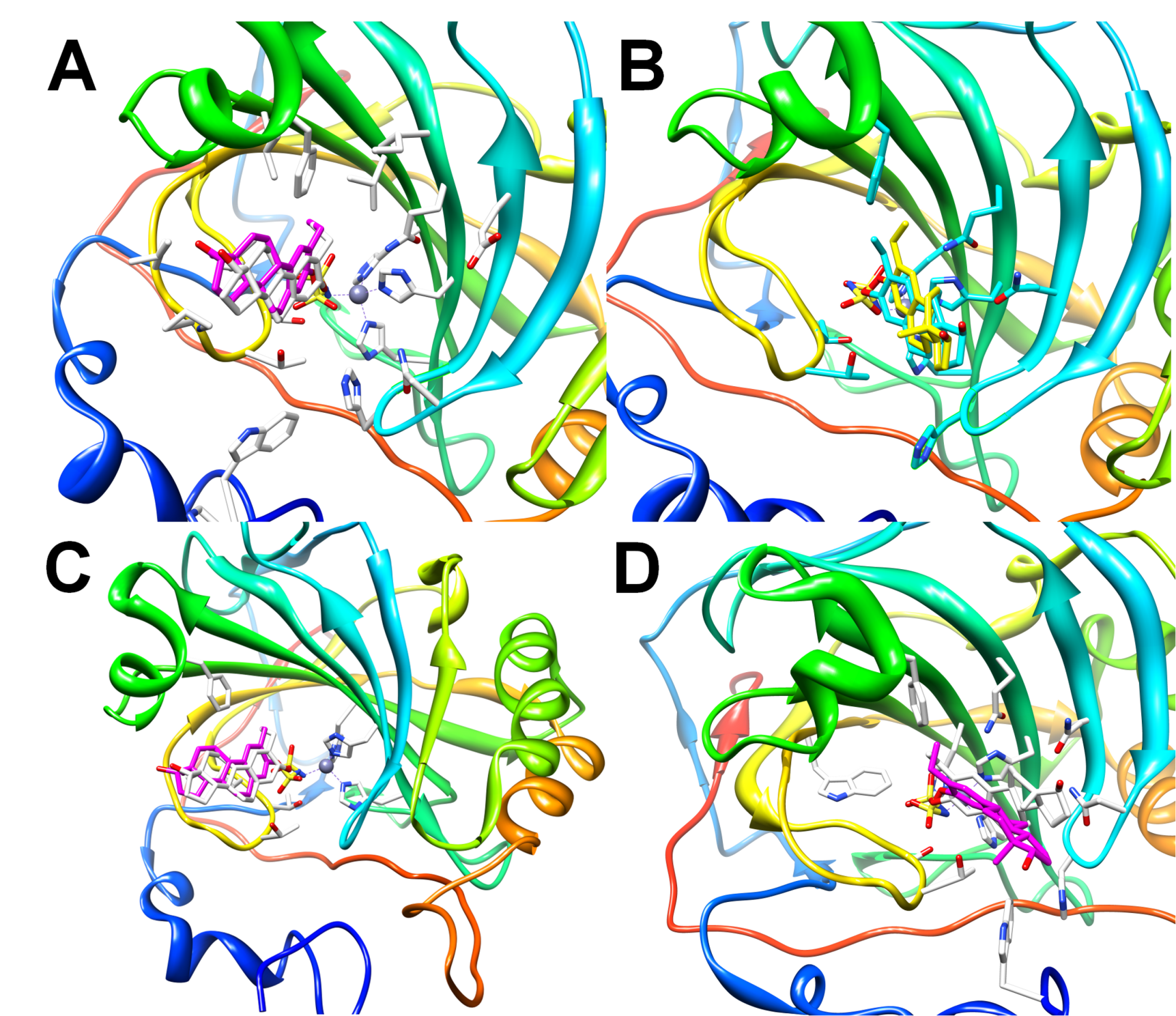

Supplement: Supporting Information S3 — Crystal structures of 2EE (white) positioned in CAII (A and C) and the CAIX mimic (B). The docking poses of 2EE into CAII (A, pink ligand) and into the CAIX mimic (B, yellow ligand) have an RMSD value of 1.332 and 1.42 respectively compared to the crystal pose. ESE-15-ol docked into CAII (C, pink ligand) show a close fit compared to the crystal pose of 2EE in CAII. (DOCX) [file pone.0052205.s003.docx]
