# Supplementary material for: In Vitro Evaluation of ESE-15-ol, an Estradiol Analogue with Nanomolar Antimitotic and Carbonic Anhydrase Inhibitory Activity
Source: PLoS One. 2012 Dec 27;7(12):e52205. doi: 10.1371/journal.pone.0052205 (PMC3531393; doi:10.1371/journal.pone.0052205)
Supplement: Supporting Information S4 — Differentially expressed genes revealed by amplified cRNA microarray and bioinformatics analyses in MDA-MB-231 cells exposed to ESE-15-ol (24 h at 50 nM). (DOCX) [file pone.0052205.s004.docx]

**Supplementary Table 1**

| **RefSeq accession numbers** | | **Agilent Reference number 44k** | **Gene Name** | | **Description** | **log M**  **(Diff expressed)**  **Exposed - Control** | ***P*-value** |
| --- | --- | --- | --- | --- | --- | --- | --- |
|  | |  |  | |  | **ESE-15-ol-treated**  **(50 nM)**  **MDA-MB-231 (24 h)** | |
| **Apoptosis, autophagy and metastasis related** | | | | | | | |
| AF100640 | | A_24_P750070 | AF100640 | | Metastasis related protein | **2.061** | 0.043 |
| NM_147129 | | A_23_P416705 | ALS2CL | | ALS2 C-terminal like | **0.767** | 0.032 |
| NM_007295 | | A_23_P207400 | BRCA1 | | Breast cancer 1, early onset | **-0.756** | 0.045 |
| NM_001731 | | A_23_P87560 | BTG1 | | B-cell translocation gene 1, anti-proliferative | **1.27** | 0.027 |
| NM_001814 | | A_23_P1552 | CTSC | | Cathepsin C | **1.083** | 0.011 |
| NM_001955 | | A_23_P214821 | EDN1 | | Endothelin 1 | **1.039** | 0.011 |
| NM_004093 | | A_24_P355944 | EFNB2 | | Ephrin-B2 | **1.065** | 0.047 |
| NM_001964 | | A_23_P214080 | EGR1 | | Early growth response 1 | **0.633** | 0.054 |
| NM_019058 | | A_23_P104318 | DDIT4 | | DNA-damage-inducible transcript 4 | **0.908** | 0.037 |
| NM_000125 | | A_23_P309739 | ESR1 | | Estrogen receptor 1 | **0.948** | 0.056 |
| NM_198897 | | A_23_P13018 | FIBP | | Fibroblast growth factor (acidic) intracellular binding protein | **-0.602** | 0.048 |
| NM_003483 | | A_23_P95930 | HMGA2 | | High mobility group AT-hook 2 | **-0.619** | 0.052 |
| NM_002128 | | A_24_P801264 | HMGB1 | | High-mobility group box 1 | **-0.758** | 0.048 |
| NM_002128 | | A_23_P99980 | HMGB1 | | High-mobility group box 1 | **-0.68** | 0.038 |
| NM_002129 | | A_23_P155765 | HMGB2 | | High-mobility group box 2 | **-0.871** | 0.021 |
| NM_198219 | | A_24_P39211 | ING1 | | Inhibitor of growth family, member 1 | **-0.681** | 0.054 |
| NM_002192 | | A_23_P122924 | INHBA | | Inhibin, beta A | **1.138** | 0.013 |
| NM_005573 | | A_23_P258493 | LMNB1 | | Lamin B1 | **-1.6** | 0.025 |
| NM_032737 | | A_23_P67725 | LMNB2 | | Lamin B2 | **-0.663** | 0.038 |
| NM_006191 | | A_24_P221123 | PA2G4 | | Proliferation-associated 2G4 | **-0.701** | 0.046 |
| NM_003579 | | A_23_P74115 | RAD54L | | RAD54-like | **-0.892** | 0.017 |
| NM_001034836 | | A_23_P141447 | RDM1 | | RAD52 motif 1 | **-0.868** | 0.044 |
| NM_006509 | | A_23_P55706 | RELB | | V-rel reticuloendotheliosis viral oncogene homolog B | **0.681** | 0.04 |
| U46752 | | A_24_P857430 | SQSTM1 | | Phosphotyrosine independent ligand p62b B-cell isoform for the Lck SH2 domain | **0.755** | 0.052 |
| NM_000548 | | A_23_P66110 | TSC2 | | Tuberous sclerosis 2 | **0.741** | 0.032 |
| NM_033285 | | A_23_P168882 | TP53INP1 | | Tumor protein p53 inducible nuclear protein 1 | **0.739** | 0.046 |
| NM_021643 | | A_23_P90696 | TRIB2 | | Tribbles homolog 2 | **0.682** | 0.055 |
| NM_145008 | | A_23_P300220 | YPEL4 | | Yippee-like 4 | **1.711** | 0.009 |
| NM_014417 | | A_23_P382775 | BBC3 | | BCL2 binding component 3 | **1.54** | 0.008 |
| **Cell cycle** | | | | | | | |
| NM_001211 | | A_23_P163481 | BUB1B | | BUB1 budding uninhibited by benzimidazoles 1 homolog beta | **-0.95** | 0.045 |
| NM_001007793 | | A_23_P202316 | BUB3 | | BUB3 budding uninhibited by benzimidazoles 3 homolog | **-0.657** | 0.045 |
| NM_005030 | | A_23_P118174 | PLK1 | | Polo-like kinase 1 | **-0.925** | 0.013 |
| NM_006622 | | A_23_P30254 | PLK2 | | Polo-like kinase 2 | **0.961** | 0.019 |
| NM_014264 | | A_23_P155969 | PLK4 | | Polo-like kinase 4 | **-0.697** | 0.06 |
| NM_001237 | | A_23_P58321 | CCNA2 | | Cyclin A2 | **-0.999** | 0.014 |
| NM_031966 | | A_23_P122197 | CCNB1 | | Cyclin B1 | **-1.173** | 0.022 |
| NM_004701 | | A_23_P65757 | CCNB2 | | Cyclin B2 | **-1.068** | 0.01 |
| NM_003858 | | A_24_P348925 | CCNK | | Cyclin K | **-0.887** | 0.045 |
| NM_001786 | | A_23_P138507 | CDC2 | | Cell division cycle 2, G1 to S and G2 to M | **-1.447** | 0.019 |
| NM_001255 | | A_23_P149200 | CDC20 | | Cell division cycle 20 homolog | **-0.994** | 0.013 |
| NM_030928 | | A_24_P176374 | CDT1 | | Chromatin licensing and DNA replication factor 1 | **-0.884** | 0.017 |
| NM_001813 | | A_23_P253524 | CENPE | | Centromere protein E | **-0.633** | 0.043 |
| NM_018451 | | A_32_P219116 | CENPJ | | Centromere protein J | **-0.656** | 0.052 |
| NM_033319 | | A_23_P126120 | CENPL | | Centromere protein L | **-0.718** | 0.044 |
| NM_018455 | | A_24_P392109 | CENPN | | Centromere protein N | **-0.718** | 0.047 |
| NM_018131 | | A_23_P115872 | CEP55 | | Centrosomal protein 55kda | **-1.094** | 0.025 |
| BC111740 | | A_24_P366107 | DNA2L | | DNA2 DNA replication helicase 2-like | **-0.714** | 0.031 |
| NM_012291 | | A_23_P32707 | ESPL1 | | Extra spindle pole bodies homolog 1 | **-0.992** | 0.021 |
| NM_181503 | | A_23_P162822 | EXOSC8 | | Exosome component 8 | **-0.799** | 0.06 |
| NM_018063 | | A_23_P12816 | HELLS | | Helicase, lymphoid-specific | **-0.603** | 0.046 |
| NM_007317 | | A_23_P54622 | KIF22 | | Kinesin family member 22 | **-0.765** | 0.037 |
| NM_012310 | | A_23_P148475 | KIF4A | | Kinesin family member 4A | **-0.835** | 0.028 |
| NM_002263 | | A_23_P133956 | KIFC1 | | Kinesin family member C1 | **-1.017** | 0.017 |
| NM_006101 | | A_23_P50108 | KNTC2 | | Kinetochore associated 2 | **-0.952** | 0.027 |
| NM_002358 | | A_23_P92441 | MAD2L1 | | MAD2 mitotic arrest deficient-like 1 | **-1.046** | 0.016 |
| NM_006739 | | A_23_P132277 | MCM5 | | MCM5 minichromosome maintenance deficient 5 | **-0.769** | 0.037 |
| NM_004153 | | A_23_P45799 | ORC1L | | Origin recognition complex | **-0.911** | 0.045 |
| NM_006879 | | A_23_P309545 | MDM2 | | Mdm2, transformed 3T3 cell double minute 2 | **1.525** | 0.009 |
| NM_002592 | | A_23_P28886 | PCNA | | Proliferating cell nuclear antigen | **-1.05** | 0.011 |
| NM_004260 | | A_23_P71558 | RECQL4 | | RECQ protein-like 4 | **-0.975** | 0.037 |
| NM_002916 | | A_23_P18196 | RFC4 | | Replication factor C | **-0.764** | 0.061 |
| NM_006306 | | A_24_P942604 | SMC1A | | Structural maintenance of chromosomes 1A | **-0.986** | 0.026 |
| NM_001042550 | | A_23_P60271 | SMC2 | | Structural maintenance of chromosomes 2 | **-1.071** | 0.019 |
| NM_020675 | | A_23_P51085 | SPC25 | | Spindle pole body component 25 homolog | **-1.089** | 0.055 |
| NM_203401 | | A_23_P200866 | STMN1 | | Stathmin 1/oncoprotein 18 | **-0.772** | 0.032 |
| **Stress related** | | | | | | | |
| NM_014685 | | A_23_P54846 | HERPUD1 | | Homocysteine-inducible, endoplasmic reticulum stress-inducible, ubiquitin-like domain member 1 | **0.7871** | 0.0613 |
| NM_002133 | | A_23_P120883 | HMOX1 | | Heme oxygenase (decycling) 1 | **0.7481** | 0.059 |
| NM_175839 | | A_23_P102731 | SMOX | | Spermine oxidase | **0.818** | 0.017 |
| NM_001024465 | | A_23_P134176 | SOD2 | | Superoxide dismutase 2 | **0.8652** | 0.0377 |
| **Kinases** | | | | | | | |
| NM_002031 | | A_23_P133665 | FRK | | Fyn-related kinase | **1.156** | 0.015 |
| NM_001005910 | | A_23_P301133 | IHPK2 | | Inositol hexaphosphate kinase 2 | **0.793** | 0.053 |
| NM_014791 | | A_23_P94422 | MELK | | Maternal embryonic leucine zipper kinase | **-1.103** | 0.021 |
| NM_017572 | | A_23_P142310 | MKNK2 | | MAP kinase interacting serine/threonine kinase 2 | **0.886** | 0.019 |
| NM_020804 | | A_23_P258088 | PACSIN1 | | Protein kinase C and casein kinase substrate in neurons 1 | **0.72** | 0.056 |
| NM_182687 | | A_24_P105102 | PKMYT1 | | Protein kinase, membrane associated tyrosine/threonine 1 | **-0.646** | 0.056 |
| NM_005030 | | A_23_P118174 | PLK1 | | Polo-like kinase 1 | **-0.925** | 0.013 |
| NM_006622 | | A_23_P30254 | PLK2 | | Polo-like kinase 2 | **0.961** | 0.019 |
| NM_014264 | | A_23_P155969 | PLK4 | | Polo-like kinase 4 | **-0.697** | 0.06 |
| NM_016203 | | A_23_P314760 | PRKAG2 | | Protein kinase, AMP-activated, gamma 2 non-catalytic subunit | **0.644** | 0.041 |
| NM_004157 | | A_23_P22214 | PRKAR2A | | Protein kinase, camp-dependent, regulatory, type II, alpha | **-0.631** | 0.045 |
| NM_145906 | | A_23_P55584 | RIOK3 | | RIO kinase 3 | **0.759** | 0.026 |
| NM_003318 | | A_23_P259586 | TTK | | TTK protein kinase | **-0.797** | 0.021 |
| NM_001826 | | A_32_P206698 | CKS1B | | CDC28 protein kinase regulatory subunit 1B | **-1.041** | 0.019 |
| NM_201444 | | A_23_P105307 | DGKA | | Diacylglycerol kinase, alpha 80kda | **0.779** | 0.061 |
| **Phosphatases** | | | | | | | |
| NM_005192 | | A_23_P48669 | CDKN3 | | Cyclin-dependent kinase inhibitor 3 | **-0.674** | 0.061 |
| NM_004417 | | A_23_P110712 | DUSP1 | | Dual specificity phosphatase 1 | **1.294** | 0.011 |
| NM_004418 | | A_24_P37409 | DUSP2 | | Dual specificity phosphatase 2 | **0.719** | 0.037 |
| NM_002004 | | A_24_P114183 | FDPS | | Farnesyl diphosphate synthase | **-0.946** | 0.018 |
| XR_018717 | | A_24_P67268 | FDPSL4 | | PREDICTED: similar to farnesyl diphosphate synthase | **-1.143** | 0.011 |
| NM_198949 | | A_23_P134295 | NUDT1 | | Nudix (nucleoside diphosphate linked moiety X)-type motif 1 | **-0.67** | 0.037 |
| NM_003713 | | A_23_P201808 | PPAP2B | | Phosphatidic acid phosphatase type 2B (PPAP2B) | **0.928** | 0.019 |
| NM_002710 | | A_23_P204423 | PPP1CC | | Protein phosphatase 1, catalytic subunit, gamma isoform | **-0.613** | 0.046 |
| NM_013239 | | A_23_P45517 | PPP2R3B | | Protein phosphatase 2 (formerly 2A), regulatory subunit B'', beta | **-0.624** | 0.049 |
| NM_002843 | | A_23_P405049 | PTPRJ | | Protein tyrosine phosphatase, receptor type, J | **0.995** | 0.016 |
| **Epigenetic and Chromatin modification** | | | | | | | |
| NM_176812 | A_23_P28969 | | CHMP4B | | Chromatin modifying protein 4B | **-1.411** | 0.053 |
| NM_001379 | A_24_P408083 | | DNMT1 | | DNA (cytosine-5-)-methyltransferase 1 | **-0.585** | 0.045 |
| NM_003642 | A_23_P339480 | | HAT1 | | Histone acetyltransferase 1 | **-0.884** | 0.039 |
| NM_021062 | A_23_P111054 | | HIST1H2BB | | Histone cluster 1, h2bb | **0.744** | 0.031 |
| NM_003524 | A_23_P366216 | | HIST1H2BH | | Histone cluster 1, h2bh | **0.697** | 0.037 |
| NM_003525 | A_23_P111041 | | HIST1H2BI | | Histone cluster 1, h2bi | **0.618** | 0.055 |
| NM_003519 | A_23_P8013 | | HIST1H2BL | | Histone cluster 1, h2bl | **0.667** | 0.036 |
| NM_003527 | A_23_P59069 | | HIST1H2BO | | Histone cluster 1, h2bo | **0.824** | 0.017 |
| NM_003537 | A_23_P93258 | | HIST1H3B | | Histone cluster 1, h3b | **-1.394** | 0.016 |
| NM_003530 | A_23_P219045 | | HIST1H3D | | Histone cluster 1, h3d | **-0.562** | 0.059 |
| NM_003534 | A_23_P42198 | | HIST1H3G | | Histone cluster 1, h3g | **-1.303** | 0.026 |
| NM_175065 | A_23_P343927 | | HIST2H2AB | | Histone cluster 2, h2ab | **-0.781** | 0.028 |
| NM_003517 | A_23_P301247 | | HIST2H2AC | | Histone cluster 2, h2ac | **-1.47** | 0.042 |
| NM_001005464 | A_24_P45651 | | HIST2H3A | | Histone cluster 2, h3a | **-0.685** | 0.031 |
| NM_001025303 | A_23_P115375 | | HIST2H3PS2 | | Histone cluster 2, H3, pseudogene 2 | **-1.442** | 0.014 |
| NM_002106 | A_23_P133146 | | H2AFZ | | H2A histone family, member Z | **-1.022** | 0.057 |
| NM_172164 | A_23_P34800 | | NASP | | Nuclear autoantigenic sperm protein (histone-binding) | **-0.757** | 0.045 |
| **Structural components** | | | | | | | |
| NM_004411 | | A_23_P93737 | DYNC1I1 | | Dynein, cytoplasmic 1, intermediate chain 1 | **0.715** | 0.041 |
| NM_015471 | | A_24_P370970 | NSL1 | | NSL1, MIND kinetochore complex component | **-0.804** | 0.021 |
| NM_145697 | | A_23_P74349 | NUF2 | | NUF2, NDC80 kinetochore complex component | **-1.196** | 0.016 |
| NM_016359 | | A_24_P416079 | NUSAP1 | | Nucleolar and spindle associated protein 1 | **-0.871** | 0.038 |
| NM_006000 | | A_23_P102109 | TUBA1 | | Tubulin, alpha 1 | **-0.568** | 0.055 |
| NM_006088 | | A_32_P187327 | TUBB2C | | Tubulin, beta 2C | **-0.782** | 0.046 |
| **Transcription factors and nuclear proteins** | | | | | | | |
| NM_005194 | | A_23_P411296 | CEBPB | CCAAT/enhancer binding protein | | **0.781** | 0.055 |
| NM_006079 | | A_23_P214969 | CITED2 | Cbp/p300-interacting transactivator, with Glu/Asp-rich carboxy-terminal domain, 2 | | **1.1** | 0.029 |
| NM_005225 | | A_23_P80032 | E2F1 | E2F transcription factor 1 | | **-0.601** | 0.045 |
| NM_024680 | | A_23_P35871 | E2F8 | E2F transcription factor 8 | | **-1.121** | 0.011 |
| NM_001968 | | A_24_P349560 | EIF4E | Eukaryotic translation initiation factor 4E (EIF4E) | | **-0.789** | 0.044 |
| NM_005252 | | A_23_P106194 | FOS | V-fos FBJ murine osteosarcoma viral oncogene | | **1.381** | 0.012 |
| NM_002158 | | A_32_P140898 | FOXN2 | Forkhead box N2 | | **-0.636** | 0.056 |
| NM_031300 | | A_23_P124559 | MXD3 | MAX dimerization protein 3 | | **-0.886** | 0.035 |
| NM_006454 | | A_23_P259490 | MXD4 | MAX dimerization protein 4 | | **0.732** | 0.032 |
| ENST00000331406 | | A_24_P911928 | MYBL1 | Myb-related protein A | | **-0.75** | 0.042 |
| NM_021005 | | A_24_P313354 | NR2F2 | Nuclear receptor subfamily 2, group F, member 2 | | **-0.967** | 0.035 |
| NM_024057 | | A_23_P48099 | NUP37 | Nucleoporin 37kda | | **-0.795** | 0.022 |
| NM_002689 | | A_23_P161615 | POLA2 | Polymerase (DNA directed), alpha 2 | | **-0.654** | 0.043 |
| NM_139215 | | A_23_P159305 | TAF15 | TAF15 RNA polymerase II | | **-1.268** | 0.032 |
| NM_003221 | | A_24_P20954 | TFAP2B | Transcription factor AP-2 beta | | **-0.553** | 0.055 |
| **RAS and RAB related proteins** | | | | | | | |
| NM_004794 | | A_23_P147025 | RAB33A | | RAB33A, member RAS oncogene family | **-1.013** | 0.016 |
| NM_006325 | | A_24_P47547 | RAN | | RAN, member RAS oncogene family | **-0.727** | 0.028 |
| NM_004165 | | A_23_P88849 | RRAD | | Ras-related associated with diabetes | **0.883** | 0.038 |
| NM_012250 | | A_23_P405761 | RRAS2 | | Related RAS viral (r-ras) oncogene homolog 2 | **0.858** | 0.019 |
| NM_013277 | | A_23_P65110 | RACGAP1 | | Rac gtpase activating protein 1 | **-0.977** | 0.06 |
| **Proteosome and ubiquitin** | | | | | | | |
| NM_003344 | | A_23_P145584 | UBE2H | | Ubiquitin-conjugating enzyme E2H | **0.781** | 0.025 |
| NM_194259 | | A_23_P152107 | UBE2I | | Ubiquitin-conjugating enzyme E2I | **-0.611** | 0.045 |
| NM_014501 | | A_32_P184933 | UBE2S | | Ubiquitin-conjugating enzyme E2S | **-0.685** | 0.035 |
| NM_014501 | | A_32_P72447 | UBE2S | | Ubiquitin-conjugating enzyme E2S | **-0.649** | 0.044 |
| NM_013282 | | A_32_P101235 | UHRF1 | | Ubiquitin-like, containing PHD and RING finger domains, 1 (UHRF1) | **-1.392** | 0.011 |
| NM_022739 | | A_23_P100754 | SMURF2 | | SMAD specific E3 ubiquitin protein ligase 2 | **0.747** | 0.053 |
| NM_003368 | | A_23_P11652 | USP1 | | Ubiquitin specific peptidase 1 | **-0.883** | 0.022 |
