# Supplementary material for: In Vitro Evaluation of ESE-15-ol, an Estradiol Analogue with Nanomolar Antimitotic and Carbonic Anhydrase Inhibitory Activity
Source: PLoS One. 2012 Dec 27;7(12):e52205. doi: 10.1371/journal.pone.0052205 (PMC3531393; doi:10.1371/journal.pone.0052205)
Supplement: Supporting Information S5 — Common differentially expressed genes revealed by amplified cRNA microarray and bioinformatics analyses in MDA-MB-231 cells exposed 24 hours to 50 nM ESE-15-ol or MCF-7 cells exposed to 1 µM 2ME. (DOCX) [file pone.0052205.s005.docx]

# Supplementary information S2: Common differentially expressed genes revealed by amplified cRNA microarray and bioinformatics analyses in MDA-MB-231 cells exposed 24 hours to 50 nM ESE-16 or MCF-7 cells exposed to 1 µM 2ME.

| **RefSeq accession numbers** | | **Agilent Reference number 44k** | **Gene Name** | **Description** | **log M**  **(Diff expressed)**  **Exposed - Control** | ***P*-value** | **log M**  **(Diff expressed)**  **Exposed - Control** | ***P*-value** |
| --- | --- | --- | --- | --- | --- | --- | --- | --- |
|  | |  |  |  | **ESE-15-ol-treated**  **(50 nM)**  **MDA-MB-231 (24 h)** | | **2ME-treated (1 µM)**  **MCF-7(24 h)** | |
| **Apoptosis, autophagy and metastasis related** | | | | | | | | |
| NM_007295 | | A_23_P207400 | BRCA1 | Breast cancer 1, early onset | **-0.756** | 0.045 | **-0.565** | 0.015 |
| NM_019058 | | A_23_P104318 | DDIT4 | DNA-damage-inducible transcript 4 | **0.908** | 0.037 | **0.752** | 0.002 |
| NM_005573 | | A_23_P258493 | LMNB1 | Lamin B1 | **-1.6** | 0.025 | **-0.761** | 0.005 |
| NM_006509 | | A_23_P55706 | RELB | V-rel reticuloendotheliosis viral oncogene homolog B | **0.681** | 0.04 | **0.533** | 0.008 |
| U46752 | | A_24_P857430 | SQSTM1 | Phosphotyrosine independent ligand p62b B-cell isoform for the Lck SH2 domain | **0.755** | 0.052 | **1.239** | 0.002 |
| NM_033285 | | A_23_P168882 | TP53INP1 | Tumor protein p53 inducible nuclear protein 1 | **0.739** | 0.046 | **0.969** | 0.019 |
| NM_145008 | | A_23_P300220 | YPEL4 | Yippee-like 4 | **1.711** | 0.009 | **1.142** | 0.002 |
| NM_014417 | | A_23_P382775 | BBC3/PUMA | BCL2 binding component 3 | **1.54** | 0.008 | **2.62** | 0.002 |
| **Cell cycle** | | | | | | | | |
| NM_001007793 | | A_23_P202316 | BUB3 | BUB3 budding uninhibited by benzimidazoles 3 homolog | **-0.657** | 0.045 | **-1.095** | 0.002 |
| NM_001237 | | A_23_P58321 | CCNA2 | Cyclin A2 | **-0.999** | 0.014 | **-0.541** | 0.014 |
| NM_031966 | | A_23_P122197 | CCNB1 | Cyclin B1 | **-1.173** | 0.022 | **-0.390** | 0.013 |
| NM_004701 | | A_23_P65757 | CCNB2 | Cyclin B2 | **-1.068** | 0.01 | **-0.472** | 0.007 |
| NM_001786 | | A_23_P138507 | CDC2 | Cell division cycle 2, G1 to S and G2 to M | **-1.447** | 0.019 | **-1.640** | 0.019 |
| NM_001255 | | A_23_P149200 | CDC20 | Cell division cycle 20 homolog | **-0.994** | 0.013 | **-0.339** | 0.016 |
| NM_001813 | | A_23_P253524 | CENPE | Centromere protein E | **-0.633** | 0.043 | **-0.539** | 0.013 |
| NM_018451 | | A_32_P219116 | CENPJ | Centromere protein J | **-0.656** | 0.052 | **-0.450** | 0.014 |
| NM_018131 | | A_23_P115872 | CEP55 | Centrosomal protein 55kda | **-1.094** | 0.025 | **-0.531** | 0.003 |
| NM_181503 | | A_23_P162822 | EXOSC8 | Exosome component 8 | **-0.799** | 0.06 | **-0.315** | 0.018 |
| NM_006739 | | A_23_P132277 | MCM5 | MCM5 minichromosome maintenance deficient 5 | **-0.769** | 0.037 | **-0.910** | 0.003 |
| NM_006879 | | A_23_P309545 | MDM2 | Mdm2, transformed 3T3 cell double minute 2 | **1.525** | 0.009 | **1.204** | 0.002 |
| NM_002592 | | A_23_P28886 | PCNA | Proliferating cell nuclear antigen | **-1.05** | 0.011 | **-0.968** | 0.008 |
| NM_004260 | | A_23_P71558 | RECQL4 | RECQ protein-like 4 | **-0.975** | 0.037 | **-0.256** | 0.017 |
| NM_002916 | | A_23_P18196 | RFC4 | Replication factor C | **-0.764** | 0.061 | **-0.829** | 0.014 |
| NM_006306 | | A_24_P942604 | SMC1A | Structural maintenance of chromosomes 1A | **-0.986** | 0.026 | **-0.770** | 0.004 |
| NM_020675 | | A_23_P51085 | SPC25 | Spindle pole body component 25 homolog | **-1.089** | 0.055 | **-0.881** | 0.002 |
| **Stress related** | | | | | | | | |
| NM_002133 | | A_23_P120883 | HMOX1 | Heme oxygenase (decycling) 1 | **0.7481** | 0.059 | **0.550** | 0.004 |
| NM_175839 | | A_23_P102731 | SMOX | Spermine oxidase | **0.818** | 0.017 | **0.713** | 0.004 |
| **Kinases** | | | | | | | | |
| NM_017572 | | A_23_P142310 | MKNK2 | MAP kinase interacting serine/threonine kinase 2 | **0.886** | 0.019 | **0.826** | 0.014 |
| NM_182687 | | A_24_P105102 | PKMYT1 | Protein kinase, membrane associated tyrosine/threonine 1 | **-0.646** | 0.056 | **-0.561** | 0.019 |
| NM_145906 | | A_23_P55584 | RIOK3 | RIO kinase 3 | **0.759** | 0.026 | **0.750** | 0.002 |
| **Phosphatases** | | | | | | | | |
| NM_004418 | | A_24_P37409 | DUSP2 | Dual specificity phosphatase 2 | **0.719** | 0.037 | **1.034** | 0.005 |
| **Epigenetic and Chromatin modification** | | | | | | | | |
| NM_003524 | A_23_P366216 | | HIST1H2BH | Histone cluster 1, h2bh | **0.697** | 0.037 | **0.568** | 0.003 |
| NM_003519 | A_23_P8013 | | HIST1H2BL | Histone cluster 1, h2bl | **0.667** | 0.036 | **0.555** | 0.004 |
| NM_003527 | A_23_P59069 | | HIST1H2BO | Histone cluster 1, h2bo | **0.824** | 0.017 | **0.563** | 0.003 |
| NM_003537 | A_23_P93258 | | HIST1H3B | Histone cluster 1, h3b | **-1.394** | 0.016 | **-0.746** | 0.002 |
| NM_003530 | A_23_P219045 | | HIST1H3D | Histone cluster 1, h3d | **-0.562** | 0.059 | **-0.765** | 0.003 |
| NM_003534 | A_23_P42198 | | HIST1H3G | Histone cluster 1, h3g | **-1.303** | 0.026 | **-0.927** | 0.009 |
| NM_175065 | A_23_P343927 | | HIST2H2AB | Histone cluster 2, h2ab | **-0.781** | 0.028 | **-0.429** | 0.010 |
| NM_003517 | A_23_P301247 | | HIST2H2AC | Histone cluster 2, h2ac | **-1.47** | 0.042 | **-0.779** | 0.006 |
| NM_001025303 | A_23_P115375 | | HIST2H3PS2 | Histone cluster 2, H3, pseudogene 2 | **-1.442** | 0.014 | **-1.244** | 0.002 |
| **Structural components** | | | | | | | | |
| NM_016359 | | A_24_P416079 | NUSAP1 | Nucleolar and spindle associated protein 1 | **-0.871** | 0.038 | **-0.345** | 0.015 |
| NM_006000 | | A_23_P102109 | TUBA1 | Tubulin, alpha 1 | **-0.568** | 0.055 | **-0.481** | 0.012 |
| NM_006088 | | A_32_P187327 | TUBB2C | Tubulin, beta 2C | **-0.782** | 0.046 | **-1.518** | 0.004 |
| **Transcription factors and nuclear proteins** | | | | | | | | |
| NM_005194 | | A_23_P411296 | CEBPB | CCAAT/enhancer binding protein | **0.781** | 0.055 | **0.474** | 0.005 |
| NM_005225 | | A_23_P80032 | E2F1 | E2F transcription factor 1 | **-0.601** | 0.045 | **-0.382** | 0.017 |
| NM_001968 | | A_24_P349560 | EIF4E | Eukaryotic translation initiation factor 4E (EIF4E) | **-0.789** | 0.044 | **-0.178** | 0.016 |
| NM_031300 | | A_23_P124559 | MXD3 | MAX dimerization protein 3 | **-0.886** | 0.035 | **-0.176** | 0.022 |
| ENST00000331406 | | A_24_P911928 | MYBL1 | Myb-related protein A | **-0.75** | 0.042 | **-0.845** | 0.006 |
| NM_021005 | | A_24_P313354 | NR2F2 | Nuclear receptor subfamily 2, group F, member 2 | **-0.967** | 0.035 | **-0.251** | 0.010 |
| **Proteosome and ubiquitin** | | | | | | | | |
| NM_003344 | | A_23_P145584 | UBE2H | Ubiquitin-conjugating enzyme E2H | **0.781** | 0.025 | **0.879** | 0.015 |
| NM_013282 | | A_32_P101235 | UHRF1 | Ubiquitin-like, containing PHD and RING finger domains, 1 (UHRF1) | **-1.392** | 0.011 | **-0.971** | 0.004 |
| NM_003368 | | A_23_P11652 | USP1 | Ubiquitin specific peptidase 1 | **-0.883** | 0.022 | **-0.668** | 0.011 |
